# Schistosomal appendicitis: a case report

**DOI:** 10.1186/s13256-024-04610-3

**Published:** 2024-06-19

**Authors:** Mohanad Khalifa, Eman Elhassan, Sawsan Abdel Rahim

**Affiliations:** 1 General Surgery Specialist, Department of Surgery, Al-Amal Hospital, Khartoum, Sudan; 2 Department of Pathology, Faculty of Medicine, University of Khartoum, Khartoum, Sudan

**Keywords:** Schistosomal appendicitis, Acute appendicitis, *Schistosoma* ova, Bilharziasis

## Abstract

**Background:**

Schistosomiasis is one of the endemic parasitic diseases in many developing countries. Despite this, appendicitis secondary to schistosomiasis is an uncommon condition even in some endemic areas. Schistosomal appendicitis, an incidentally discovered appendicitis associated with schistosomiasis histological findings, affects young males predominantly. Timely diagnosis and treatment, including appendectomy and anti-helminthic therapy, are crucial.

**Case report:**

A 24-year-old Sudanese male patient presented with abdominal pain. Diagnosed with acute appendicitis, he underwent appendectomy, revealing appendix inflammation with *Schistosoma* ova in histopathology. Abdominal ultrasound detected no complications. Weakly positive *Schistosoma* serology was noted, but stool and urine analysis showed no infection evidence. Prescribed praziquantel, patient had 3-year post-op follow-up without complications.

**Conclusions:**

This case report underscores the significance of including schistosomiasis in the differential diagnosis of appendicitis, particularly in regions where the disease is endemic. It underscores the necessity of histopathological evaluations for accurate diagnosis, emphasizing the potential implications for clinical practice in similar settings.

## Introduction

Schistosomal appendicitis is a rare condition that occurs when the appendix is affected by schistosomiasis. This parasitic infection is more commonly seen in endemic areas but can also be encountered in developed countries due to global travel and migration patterns [1].

The prevalence of schistosomal appendicitis varies globally, with studies reporting a range of 1.31–3.2%. The condition predominantly affects young males, with a male-to-female ratio of 9:1 [2, 3].

Medical practitioners should consider the possibility of schistosomal appendicitis when diagnosing acute appendicitis in patients. Timely and appropriate treatment, including appendectomy and anti-helminthic therapy, is essential for addressing acute appendicitis and averting potential long-term consequences of schistosomiasis. This study recounts the experience of a 24-year-old male in Sudan who presented with acutea appendicitis, and subsequent histopathological examination revealed the presence of *Schistosoma* incidentally.

The case of schistosomal appendicitis is important because it sheds light on a rare but serious condition caused by a parasitic infection. It teaches doctors about how to recognize and treat this condition, which can be tricky because it is not common everywhere. This ultimately helps improve outcomes for those affected.

## Case report

The patient was a 24-year-old African male from Sudan, residing in Khartoum, the capital. He has not traveled outside the country but occasionally visits his father’s farm, where he swims in the stream. He has no significant medical history but has a family history of controlled diabetes in his father and controlled bronchial asthma in his mother. This patient presented with abdominal pain lasting for 3 days. The pain initially started in the umbilicus and then shifted to the right iliac fossa. The patient also experienced vomiting and attempted self-treatment at home with herbal medications for 3 days; however, the symptoms continued getting worse, so he sought medical advice on the third day. He arrived at the Emergency department with typical features of acute appendicitis, with an Alverado score of 8 (scores range from 0 to 10, with higher scores suggesting a higher likelihood of appendicitis. A score of 7 or above typically indicates a high probability of appendicitis) [5] Consequently, he underwent an appendectomy on the same day.

**Fig. 1:**
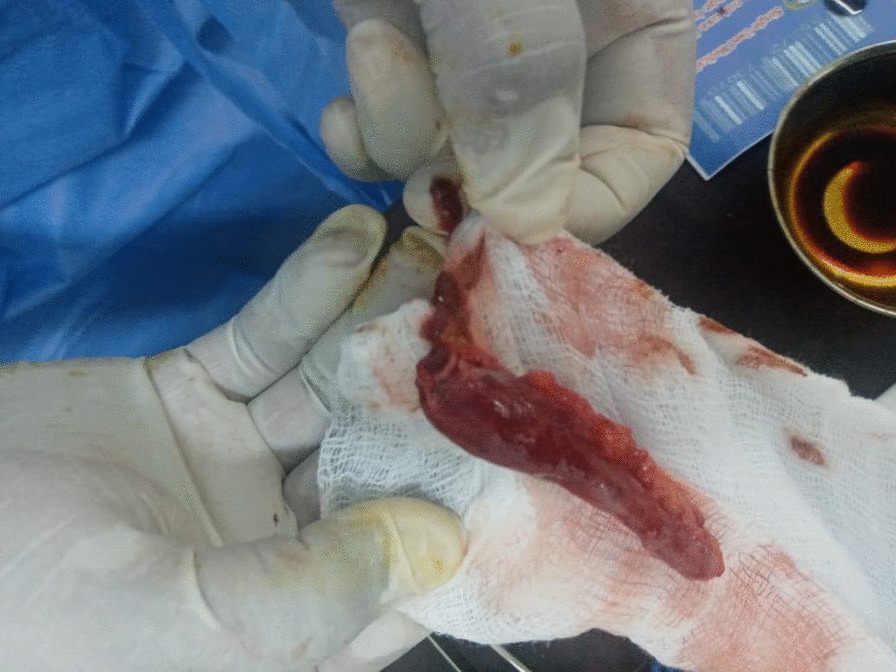
Removed appendix

**Fig. 2:**
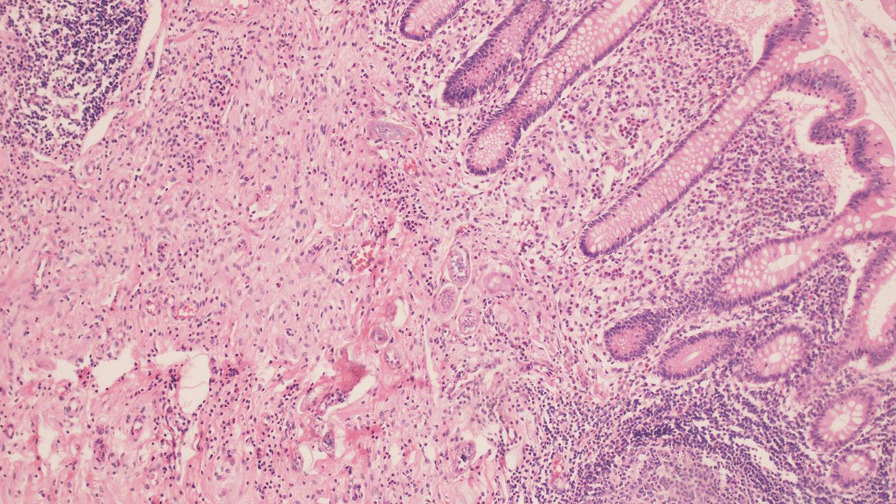
Multiple calcified and viable *Schistosoma* ova surrounded by moderate inflammation in submucosa of appendix

**Fig. 3:**
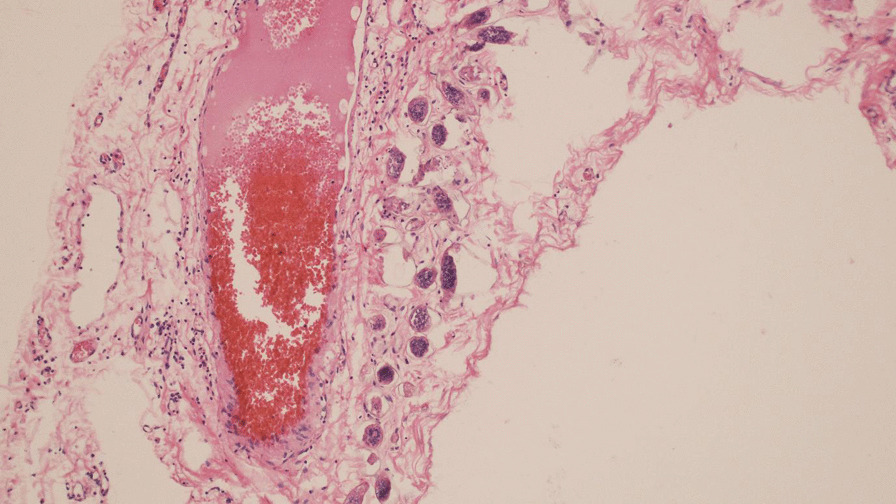
Serosal involvement by viable *Schistosoma* ova

**Fig. 4:**
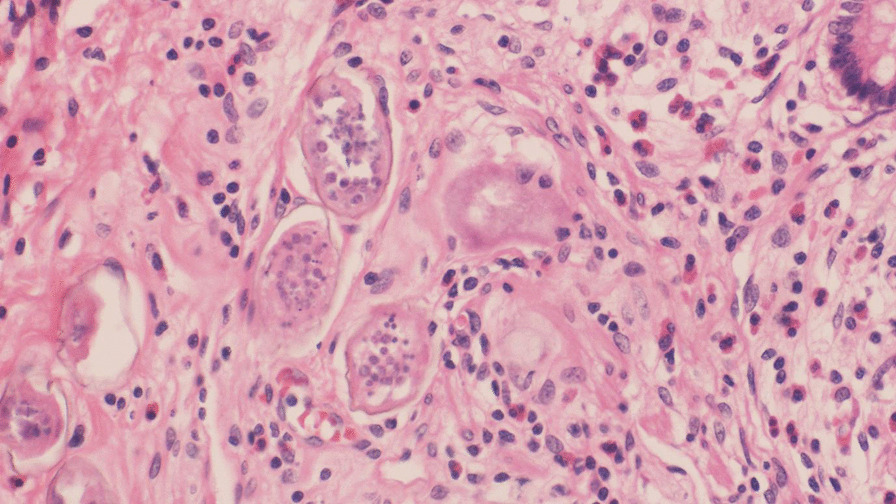
Higher power shows marked inflammatory infiltrate composed mainly of eosinophils surrounding *Schistosoma* ova

During the procedure, inflammation was observed in the appendix, with no other significant intraoperative findings (Fig. 1). The histopathological examination revealed the presence of *Schistosoma* ova surrounded by moderate inflammation in the submucosa of the appendix (Figs. 2, 3, 4. An abdominal ultrasound was performed after 2 weeks from surgery to check for complications related to chronic infection, such as hepatosplenic disease or urinary findings, and showed no abnormalities.

Despite weakly positive *Schistosoma* serology and negative findings in stool, urine analyses, and imaging for schistosomiasis in the liver, lung, or bladder, it remains crucial for patients with parasitic infestations to undergo appropriate antiparasitic treatment (praziquantel dosing according to body weight) before hospital discharge. This is because appendectomy only addresses the consequence and not the root cause of the disease. Following the surgery, the patient had a clear follow-up for the next 3 years with out any new clinical, laboratory, or radiology findings of the disease.

## Discussion

Schistosomiasis is one of endemic parasitic disease in many developing countries [4]. Despite this, appendicitis secondary to schistosomiasis is an uncommon condition even in endemic areas.

A systematic review of the literature demonstrated a total prevalence of 1.3%, while stratification by continents revealed a prevalence of 2.8% in Africa compared with 0.5% in the Middle East [2]. By contrast, studies in non-endemic countries reveal a prevalence rate of 0.1–0.2% [6].

In Sudan, schistosomiasis is a major health problem, especially among school-aged children. The prevalence of schistosomiasis-related appendicitis in Sudan is not explicitly mentioned. However, a nationwide survey in Sudan estimated the prevalence of schistosomiasis to be 5.2% for *Schistosoma haematobium* and 0.06% for *Schistosoma mansoni* [7].

The pathophysiological mechanisms of schistosomal appendicitis are not fully understood. Ischemic changes caused by egg emboli constitute a possible element. However, there is evidence suggesting that the immune response of the host to *Schistosoma* eggs deposited in the appendix may play a role in generating granulomas. This reaction may diminish mucosal immunity, thus leading to elevating vulnerability to bacterial infections and the onset of acute appendicitis [8–10].

Clinicians and pathologists should be aware of schistosomal appendicitis, particularly in endemic areas, as it can present with features of intestinal obstruction and acute appendicitis.

Clinical presentation of schistosomal appendicitis is similar to other causes of acute appendicitis, and routine investigations provide limited additional information. Diagnosis involves serology, polymerase chain reaction assays, and histopathological confirmation with the presence of *Schistosoma* ova and granulomatous inflammation [1].

Regardless of the pathophysiology, acute appendicitis represents a surgical emergency. Appendectomy is the standard treatment for acute appendicitis [11]. The removal of the inflamed appendix is to prevent complications such as perforation.

Antiparasitic therapy with praziquantel is the first-line treatment for schistosomiasis. It has been shown to be effective in reducing the prevalence and intensity of the parasite infection [12].

It is essential that patients with parasitic infestation receive the appropriate antiparasitic treatment before being discharged from hospital because the appendectomy is treated as a consequence and not the root of the disease. Antiparasitic therapy with praziquantel is used to treat the underlying schistosomiasis infection and prevent further complications [13, 14]. Clinicians and pathologists should be aware of schistosomal appendicitis, particularly in regions where it is endemic, to facilitate early diagnosis and appropriate treatment.

## Conclusion

In areas where schistosomiasis is prevalent as in Sudan, it is advisable to conduct histopathological assessments on surgically excised appendices. Surgeons should maintain a high index of suspicion, especially in patients with a travel history to endemic regions. They should be vigilant for atypical symptoms and signs such as prolonged symptoms, eosinophilia, or chronic abdominal pain. Early consideration of schistosomiasis can prompt timely testing by serology, polymerase chain reaction assays, and histopathological confirmation with the presence of *Schistosoma* ova and early intervention, helping prevent complications.

## Data Availability

The data are available in the medical records of the Surgery department at Al-Amal Hospital and the Prof. Abdelsater Histopathology Center, Khartoum, Sudan.
